# A Low-Cost Facial and Dental Nerve Regional Anesthesia Task Trainer

**DOI:** 10.21980/J8RP9Q

**Published:** 2021-04-19

**Authors:** Andrew Eyre, Valerie Dobiesz

**Affiliations:** * Brigham and Women’s Hospital/Harvard Medical School, Department of Emergency Medicine, Boston, MA; ^ STRATUS Center for Medical Simulation/Brigham and Women’s Hospital, Department of Emergency Medicine, Boston, MA

## Abstract

**Audience:**

This facial and dental regional anesthesia task trainer is designed for teaching emergency medicine (EM) residents and medical students; however, it can be used by other specialties including plastic surgery, otolaryngology, oral surgery, and dentistry.

**Introduction:**

While the opioid epidemic remains a public health crisis, emergency departments (EDs) continue to treat patients who require painful facial procedures or who present with severe dental pain. There is increasing interest and renewed use of regional anesthesia for procedural anesthesia and as an effective non-opioid analgesic. Although many nerve blocks are now being taught using ultrasound guidance, regional anesthesia of the face and mouth is still performed using landmark-based techniques. To date, there are no commercially available task trainers for teaching regional anesthesia of the face and mouth. Therefore, a low-cost, feedback-enhanced, partial task trainer was created for teaching regional anesthesia of the supraorbital, infra-orbital, mental, and inferior alveolar nerves.

**Educational Objectives:**

By the end of this educational session, learners should be able to:

1. Describe and identify relevant anatomy for supra-orbital, infra-orbital, mental, and inferior alveolar nerves.
2. Successfully demonstrate supra-orbital, infra-orbital, mental, and inferior alveolar nerve blocks using a partial task trainer.

**Educational Methods:**

Using inexpensive and commonly found materials, we were able to successfully modify an existing airway task trainer in order to create a feedback-enhanced partial task trainer for teaching supra-orbital, infra-orbital, mental, and inferior alveolar regional anesthesia. When the needle is inserted in the correct nerve location by the learner, the task trainer provides positive feedback in the form of an audible alert. This innovative task trainer has been used to teach post graduate year (PGY) 1–4 resident learners as part of a standard emergency medicine residency didactics curriculum. After a brief introductory didactic session, participants are given the opportunity for hands-on skills practice using the task trainer under faculty supervision.

**Research Methods:**

An existing airway task trainer was successfully modified in order to create a feedback-enhanced, partial task trainer for teaching supra-orbital, infra-orbital, mental, and inferior alveolar regional anesthesia. Learners were asked to complete a post-session survey to assess the educational value of the station and the task trainer.

**Results:**

Twenty-one residents (10 PGY-1, 9 PGY-3, 2 PGY-4) participated in a didactic session and all completed a brief post-session survey. Many participants (N=10, 48%) had never previously performed any of these nerve blocks. On average, participants rated their comfort performing these specific nerve blocks before the session to be 1.96 on a 5-point Likert-scale (where 1=not at all comfortable and 5=extremely comfortable). Following the session, participants’ comfort level increased to 3.67 on the same scale. Participants rated the usefulness of the feedback-enhanced task trainer to be a 4.71 on a 5-point scale (where 1=not at all useful and 5=extremely useful).

**Discussion:**

Using inexpensive and commonly available materials, we were able to successfully modify an existing airway task trainer in order to create a feedback-enhanced partial task trainer for teaching regional anesthesia of the face and mouth. Learners reported that the educational session greatly increased their confidence in performing supra-orbital, infra-orbital, mental, and inferior alveolar nerve blocks. Additionally, they found the feedback-enhanced partial task trainer to be extremely helpful for teaching appropriate landmark identification. Our model was successfully used to teach facial and dental nerve block techniques which are able to provide both procedural anesthesia and non-opioid analgesia. Future studies could investigate whether this educational session and model leads to increased competence and/or increased performance of facial nerve blocks in the clinical setting.

**Topics:**

Nerve blocks, regional anesthesia, dental emergencies, facial trauma.

## USER GUIDE

**Table t1-jetem-6-2-i1:** 

**List of Resources:**	
Abstract	1
User Guide	3


[Table t1-jetem-6-2-i1]

**Learner Audience:**

Interns, Junior Residents, Senior Residents

**Time Required for Implementation:**

The task trainer can be assembled in approximately 30 minutes if a preexisting airway task trainer is available for modification. We recommend each educational session, although flexible, be scheduled for 45 minutes to allow for a brief didactic session and demonstration followed by hands-on practice. Given that learners may not regularly perform all of these specific nerve blocks in the clinical setting, we recommend including this educational session on an annual or bi-annual basis as part of a standardized didactics curriculum.

**Recommended Number of Learners per Instructor:**

We recommend a ratio of 1 task trainer for every 4 to 5 learners with a maximum instructor to learner ratio of 1:10.

**Topics:**

Nerve blocks, regional anesthesia, dental emergencies, facial trauma.

**Objectives:**

By the end of the educational session, learners should be able to:

1. Describe and identify relevant anatomy for supraorbital, infra-orbital, mental, and inferior alveolar nerves.
2. Successfully demonstrate supra-orbital, infraorbital, mental, and inferior alveolar nerve blocks using a partial task trainer.

### Linked objectives and methods

Simulation-based medical education is a widely accepted, effective, and popular modality for teaching both cognitive and procedural skills. Built upon a wide array of educational theories and literature, simulation is an active teaching strategy that not only enhances learning by providing a contextual framework, but also allows learners to process and use knowledge in an engaging way. As described by Bloom’s Taxonomy^1^ learners can acquire and process information at differing levels. While lectures primarily transmit information and ask learners to remember or recall new information, simulation-based medical education allows learners to demonstrate the acquisition as well as the application of knowledge, both of which represent higher levels of understanding on Bloom’s Taxonomy.

Simulation also provides learners with a consistent and safe environment for practice and learning. Whereas the clinical environment is highly variable and instructors cannot guarantee that learners will encounter certain pathology or cases, simulation-based medical education can ensure that all learners are exposed to requisite experiences. Additionally, simulation provides learners with the opportunity to learn and practice skills without introducing additional risks to patients or themselves.

While simulation can be used to teach a wide variety of skills and concepts, it is the ideal modality for teaching low frequency or high-risk procedures. Given the importance of providing anesthesia and appropriate analgesia, coupled with the numerous indications for blocks of the supra-orbital, infra-orbital, mental, and inferior alveolar nerves, it is critical that emergency physicians gain experience and comfort performing these blocks. By integrating a positive-feedback mechanism, our task trainer allows learners to perform deliberate practice, either independently or with faculty supervision, while ensuring that they correctly perform the procedure.

### Recommended pre-reading for instructor

- Moskovitz J, Sabatino F. Regional nerve blocks of the face. Emerg Med Clin North Am. 2013;31(2):517–527. doi:10.1016/j.emc.2013.01.003
- PEM Pearls: Regional Facial Nerve Blocks: https://www.aliem.com/pem-pearls-regional-facial-nerve-blocks/
- EMDocs: Facial Nerve Blocks http://www.emdocs.net/facial-nerve-blocks/
- Downeast EM: Facial Nerve Blocks https://www.downeastem.org/nerve-blocks-of-the-face-and-mouth

### Learner responsible content (LRC)

- PEM Pearls: Regional Facial Nerve Blocks: https://www.aliem.com/pem-pearls-regional-facial-nerve-blocks/
- EMDocs: Facial Nerve Blocks http://www.emdocs.net/facial-nerve-blocks/
- Downeast EM: Facial Nerve Blocks https://www.downeastem.org/nerve-blocks-of-the-face-and-mouth

### Implementation Methods

The task trainer must be created and tested prior to the educational session to ensure that all electrical connections are working properly and safely secured. We recommend that the instructor spend the first 15 minutes of the 45-minute session reviewing the common indications for these specific nerve blocks, relevant anatomy and landmarks, complications, anesthetic dosing, and appropriate insertion techniques. The instructor should then demonstrate each of these nerve blocks on the task trainer. Following the introduction, learners should practice each of these nerve blocks on the feedback-enhanced task trainer, with the instructor providing feedback and answering questions. At the conclusion of the educational session, the instructor provides a brief recap of the high-yield learning points, as guided by the learning objectives.

### List of items required to replicate this innovation

The feedback-enhanced task trainer is made by using inexpensive and commonly available materials that modify an existing airway task trainer. Many commercially available airway task trainers can be substituted for the base.

1. Airway Trainer: https://www.laerdal.com/us/doc/92/Laerdal-Airway-Management-Trainer
2. Roll Standard Kitchen Aluminum Foil: https://www.amazon.com/365-Everyday-Value-Aluminum-Foil/
3. 20-inch Double Ended Test Lead Jumper Wire with Alligator Clips: https://www.walmart.com/ip/Unique-Bargains-10-Pcs-20-Double-ended-Alligator-Clips-Test-Lead-Jumper-Wire-50cm-5-Color/45586113
4. 3-24v Piezo Electric Buzzer: (https://www.amazon.com/Electric-Buzzer-Physics-Circuits-Continuous/
5. Standard 9 Volt Battery
6. 3/5 inch Electrical Tape: (https://www.amazon.com/Electrical-Waterproof-Retardant-Temperature-Resistant/
7. 10cc Syringe: https://www.vitalitymedical.com/10-ml-syringe-without-needle.html
8. 25g Needle: https://www.buyemp.com/product/exel-hypodermic-syringe-needles
9. 30g Copper Electric Hookup Wire: https://www.amazon.com/StrivedayTM-Flexible-Silicone-electronic-electrics/
10. Wire Cutters: https://www.amazon.com/Dykes-Cutter-Diagonal-Cutting-Pliers/

### Approximate cost of items to create this innovation

This innovative, feedback-enhanced task trainer is created by modifying an existing airway trainer using materials that are commonly available and inexpensive. The items needed for the necessary modifications cost approximately $10 to $15 (US dollars), depending on local purchasing agreements and existing simulation resources. However, the overall task trainer will cost more if a new airway task trainer is purchased separately.

### Detailed methods to construct this innovation

1. Identify the airway trainer to modify.[Fig f1-jetem-6-2-i1]
  **Figure f1-jetem-6-2-i1:**
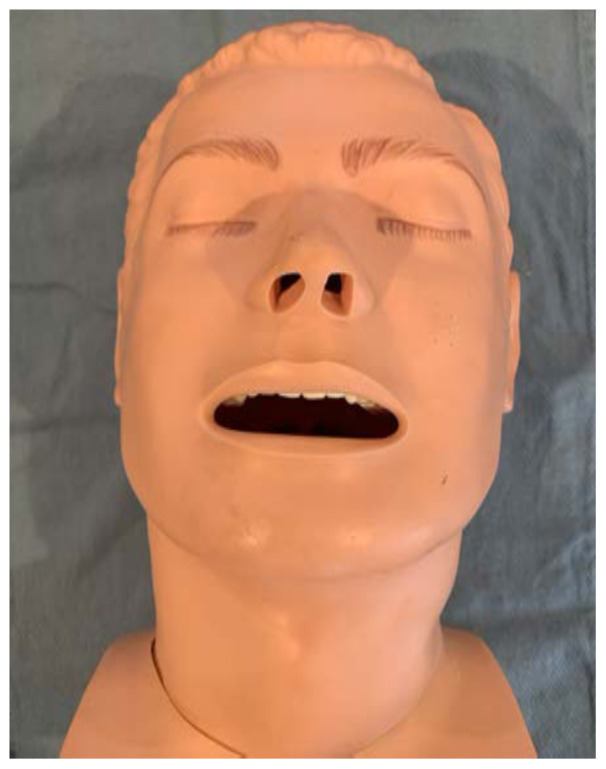
2. Remove the simulated skin that covers the facial structures.
3. Identify the proper anatomical target zones for the nerve blocks.
4. Cut a 2’ long strand of electric wire for each target zone you wish to create (supra-orbital, infra-orbital, mental, and inferior alveolar). Double this number if you would like bilateral target zones. You may wish to use one color electric wire for the right-sided target zones and another color for the left-sided target zones.[Fig f2-jetem-6-2-i1]
  **Figure f2-jetem-6-2-i1:**
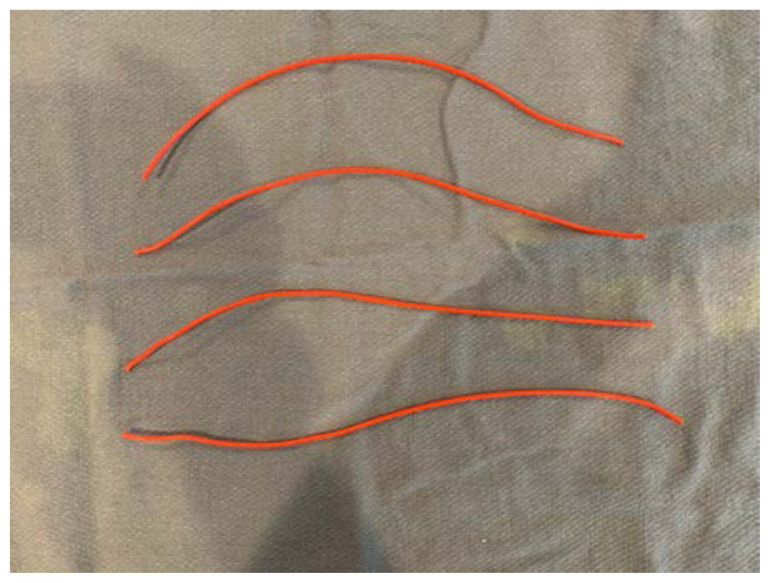
5. For each strand of electric wire, use a wire cutter to remove 1cm of insulation from each end.[Fig f3-jetem-6-2-i1]
  **Figure f3-jetem-6-2-i1:**
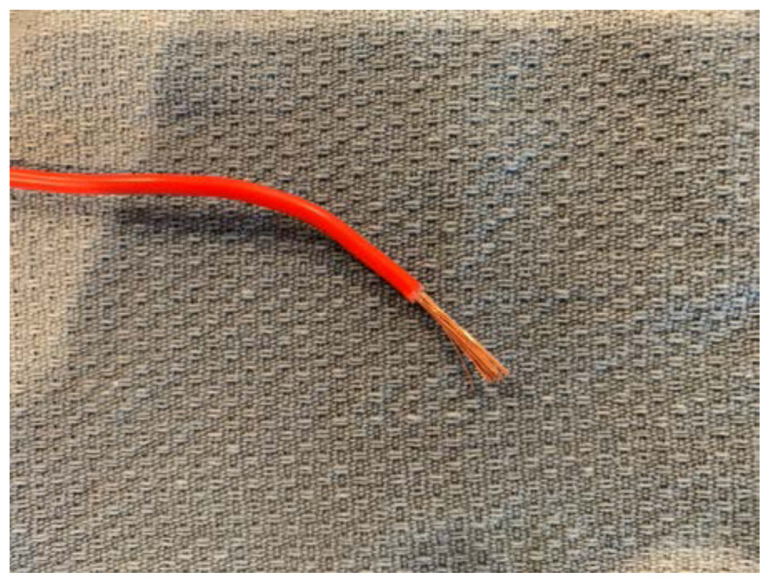
6. For each target zone, mold a sheet of aluminum foil around the exposed end of a strand of electric wire.[Fig f4-jetem-6-2-i1]
  **Figure f4-jetem-6-2-i1:**
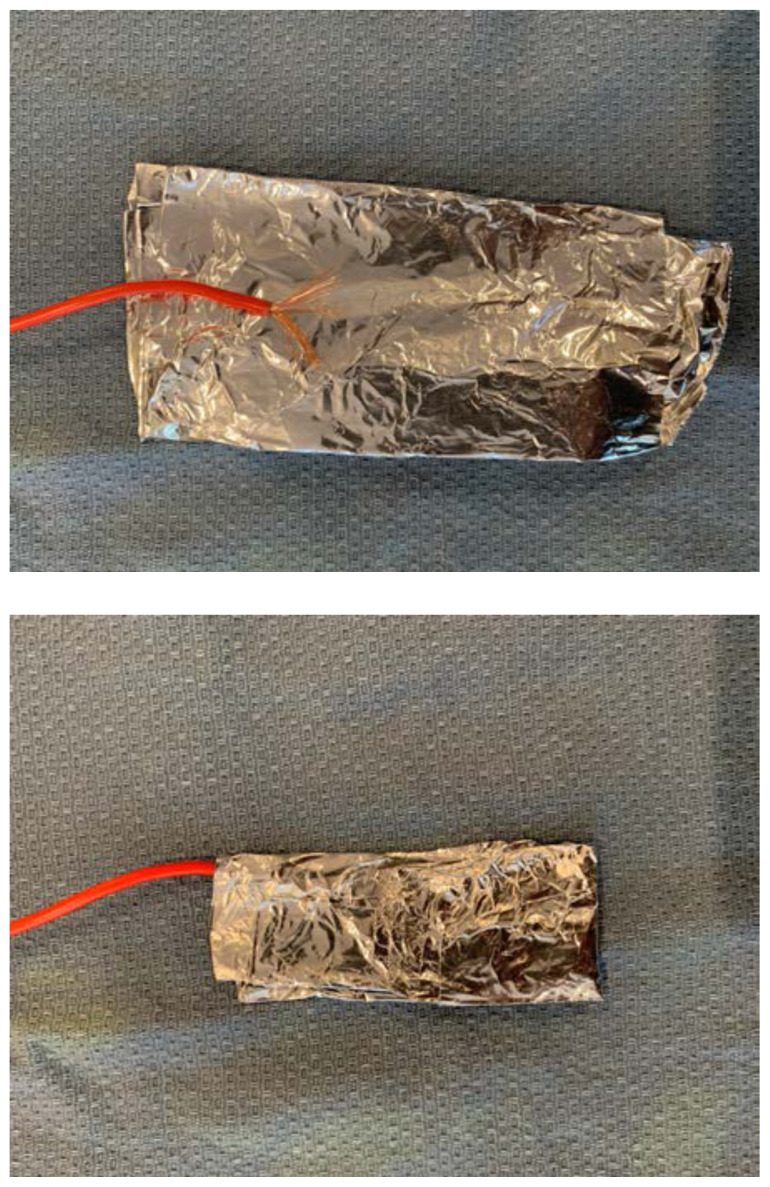
7. Mold and attach the aluminum foil target to the correct anatomical location on the simulated skull using electrical tape.
8. Repeat steps 5–7 for each target zone. The size of the aluminum foil target can be adjusted for each anatomical location and for the desired level of difficulty. On average, the foil target should be approximately 2–3cm by 1cm, however smaller targets can be used to increase the level of difficulty.[Fig f5-jetem-6-2-i1]
  **Figure f5-jetem-6-2-i1:**
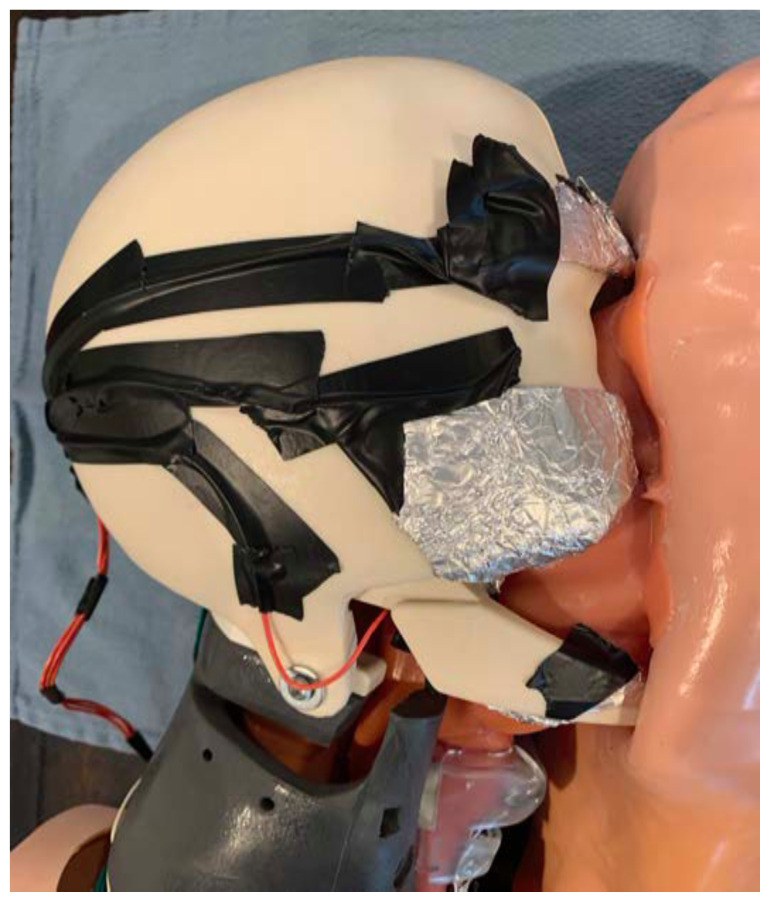
9. Secure the electric wire strands to the simulated skull using electrical tape. It is optimal to tape the right sided strands together into one “cord” and the left sided strands together into a separate “cord.”[Fig f6-jetem-6-2-i1]
  **Figure f6-jetem-6-2-i1:**
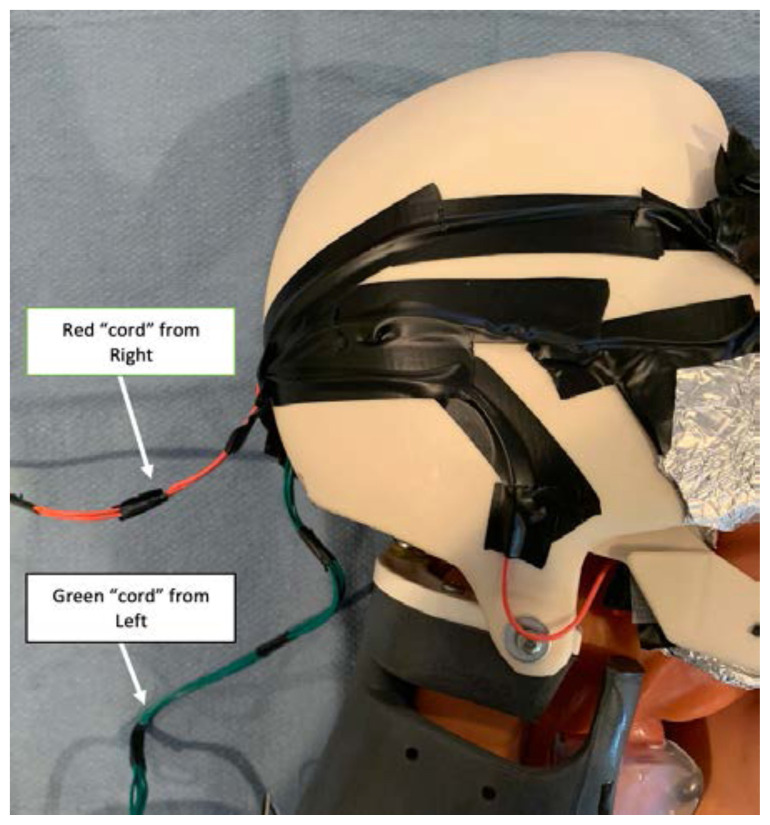
10. Bring each “cord” to the back of the simulated skull, or wherever they will exit the skin of the airway trainer you selected[Fig f7-jetem-6-2-i1]
  **Figure f7-jetem-6-2-i1:**
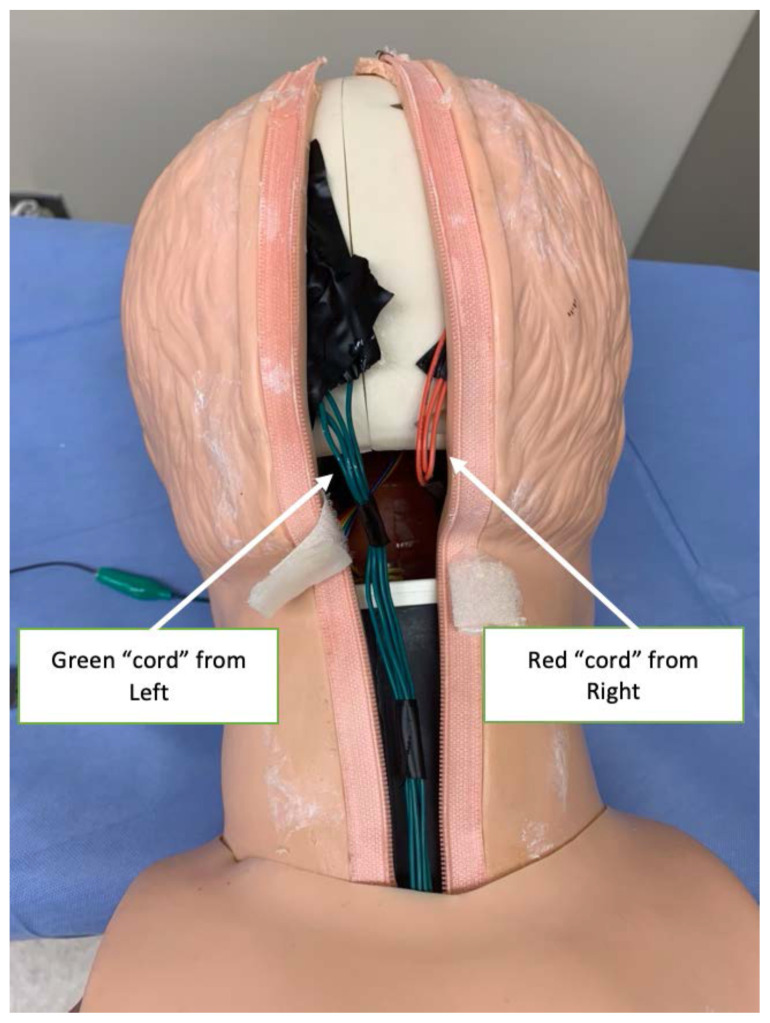
11. Carefully return the simulated skin to its original position, bringing it up and over the target zones. Alternatively, you may wish to leave this until the final step to ensure that all electrical connections are working properly.[Fig f8-jetem-6-2-i1]
  **Figure f8-jetem-6-2-i1:**
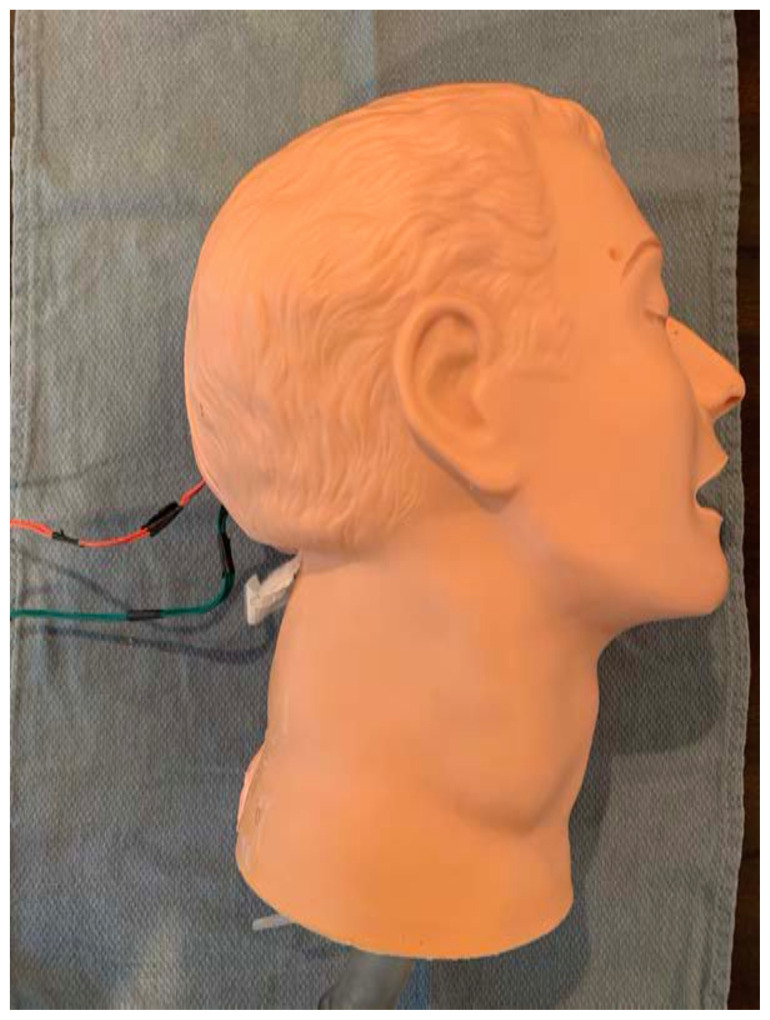
12. Twist the exposed distal ends of the electrical wire together[Fig f9-jetem-6-2-i1]
  **Figure f9-jetem-6-2-i1:**
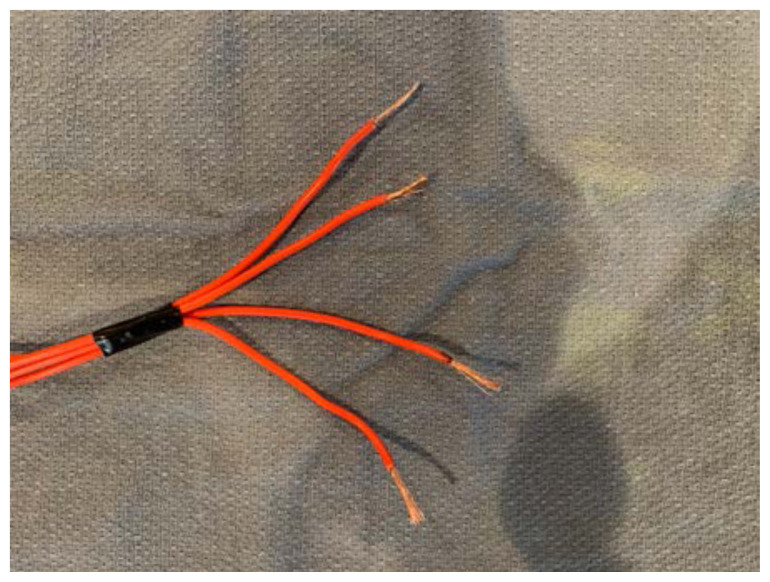

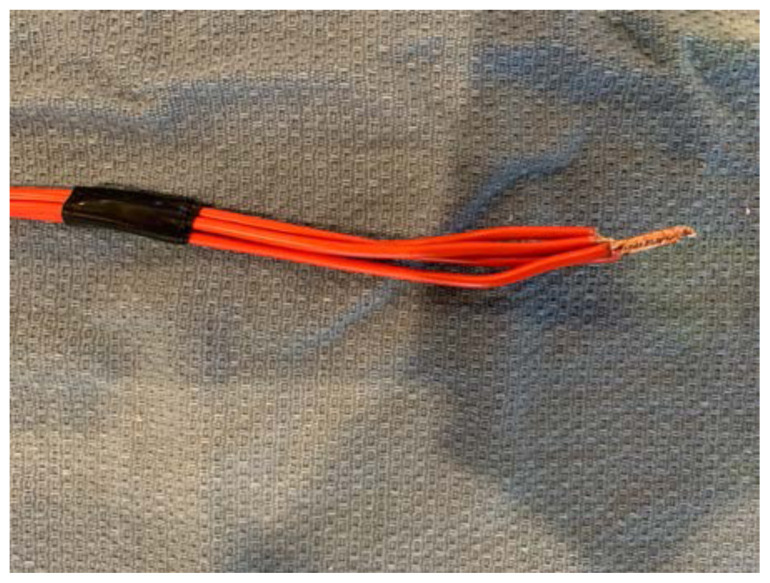
13. Attach one end of an alligator clip #1 to this bundle of exposed ends. Secure with electrical tape.[Fig f10-jetem-6-2-i1]
  **Figure f10-jetem-6-2-i1:**
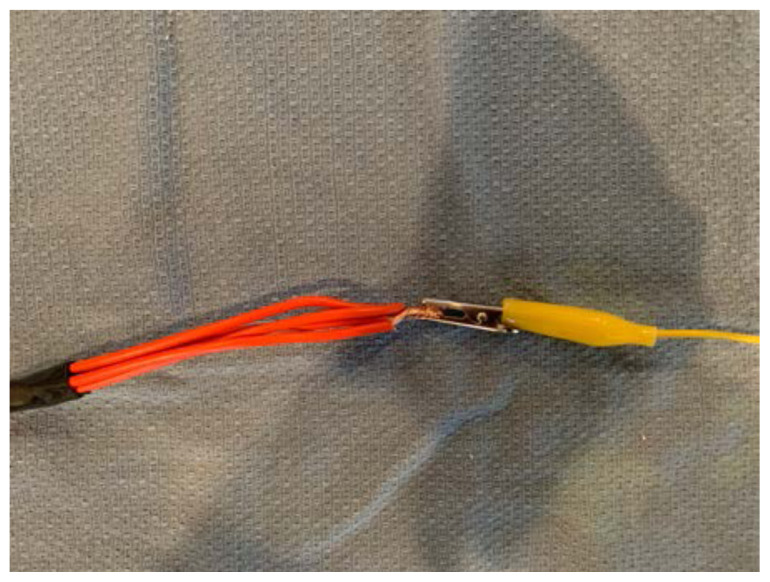
14. Attach the other end of alligator clip #1 to one end of the piezo electric buzzer.[Fig f11-jetem-6-2-i1]
  **Figure f11-jetem-6-2-i1:**
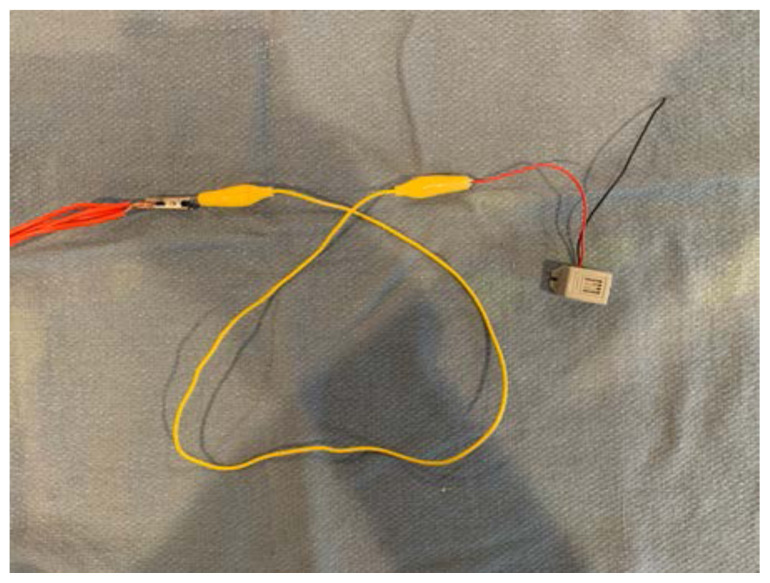
15. Attach one end of alligator clip #2 to the other end of the piezo electric buzzer.[Fig f12-jetem-6-2-i1]
  **Figure f12-jetem-6-2-i1:**
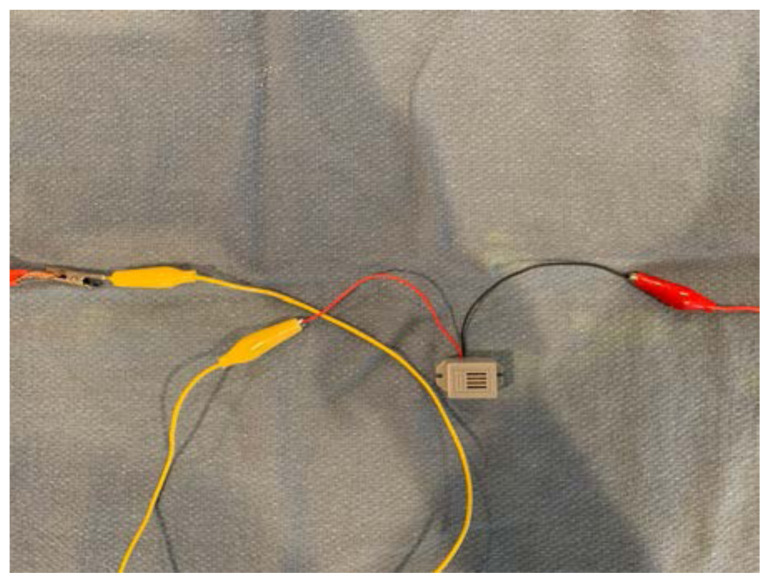
16. Attach the other end of alligator clip #2 to one electrode of the 9V battery.[Fig f13-jetem-6-2-i1]
  **Figure f13-jetem-6-2-i1:**
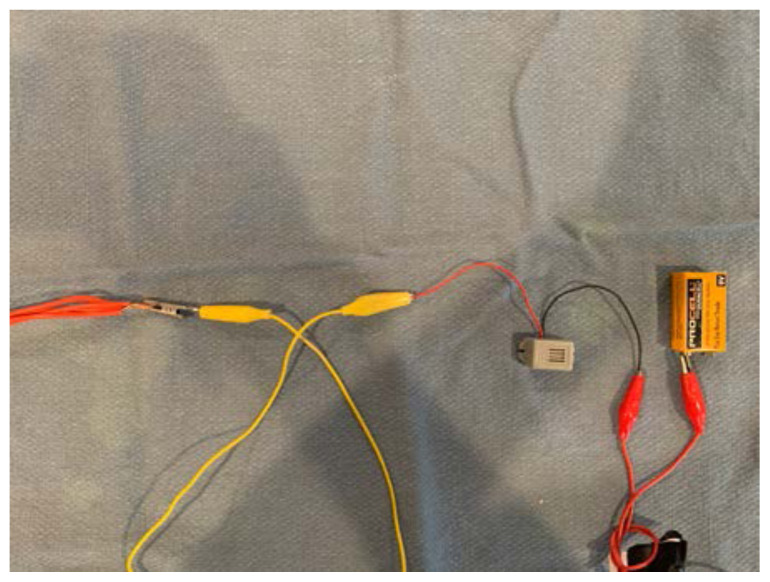
17. Attach one end of alligator clip #3 to the other electrode of the 9V battery.[Fig f14-jetem-6-2-i1]
  **Figure f14-jetem-6-2-i1:**
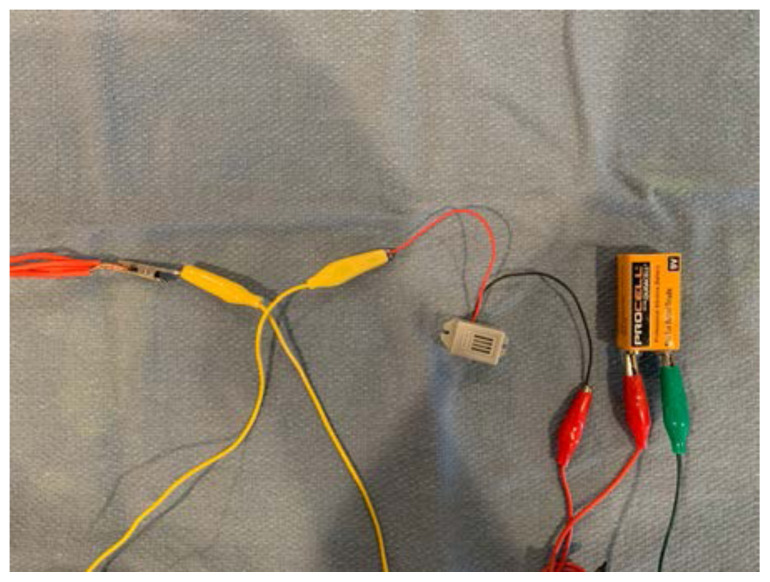
18. Connect the needle and syringe.
19. Either connect the other end of alligator clip #3 directly to the needle, or you may connect it to another strand of electrical wire that is then connected and secured to the needle after the distal edges of insulation have been removed.[Fig f15-jetem-6-2-i1]
  **Figure f15-jetem-6-2-i1:**
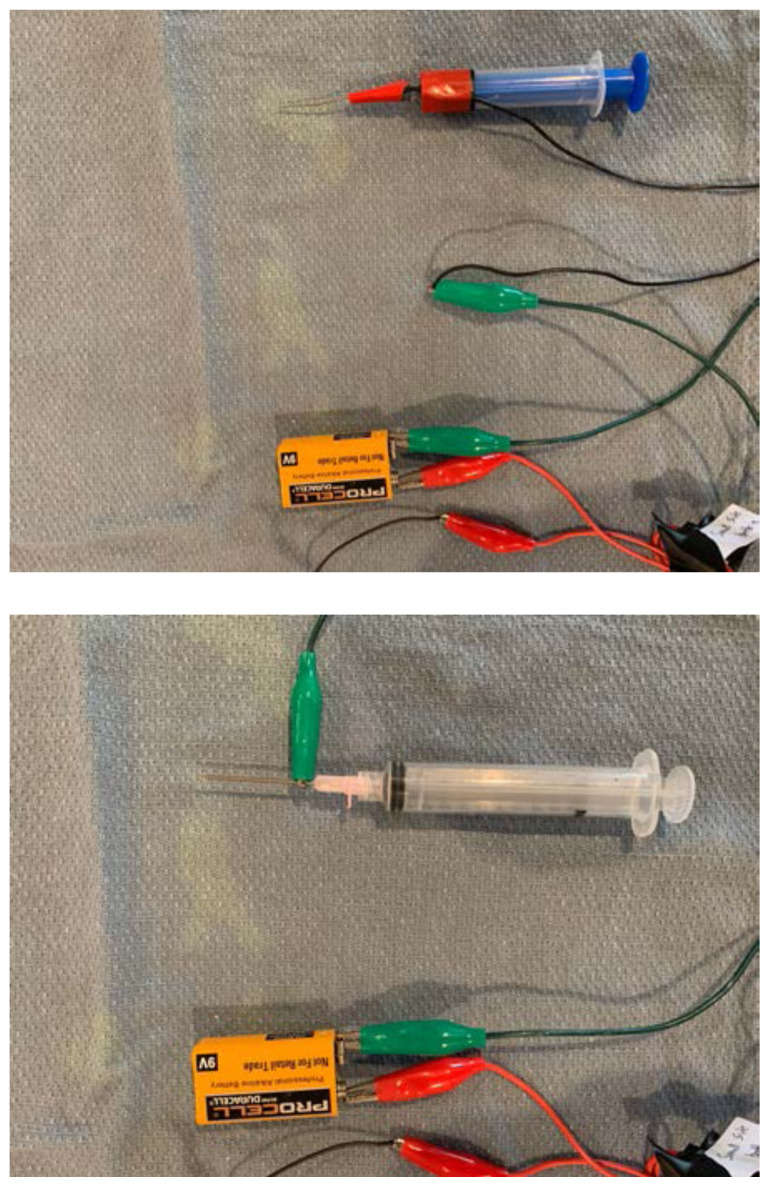
20. Test the model by inserting the needle into one of the target zones. You may need to experiment and adjust the connections of the buzzer and/or battery to ensure that current is flowing in the correct directions.
21. Reassemble the airway trainer to hide unnecessary electrical components.[Fig f16-jetem-6-2-i1]
  **Figure f16-jetem-6-2-i1:**
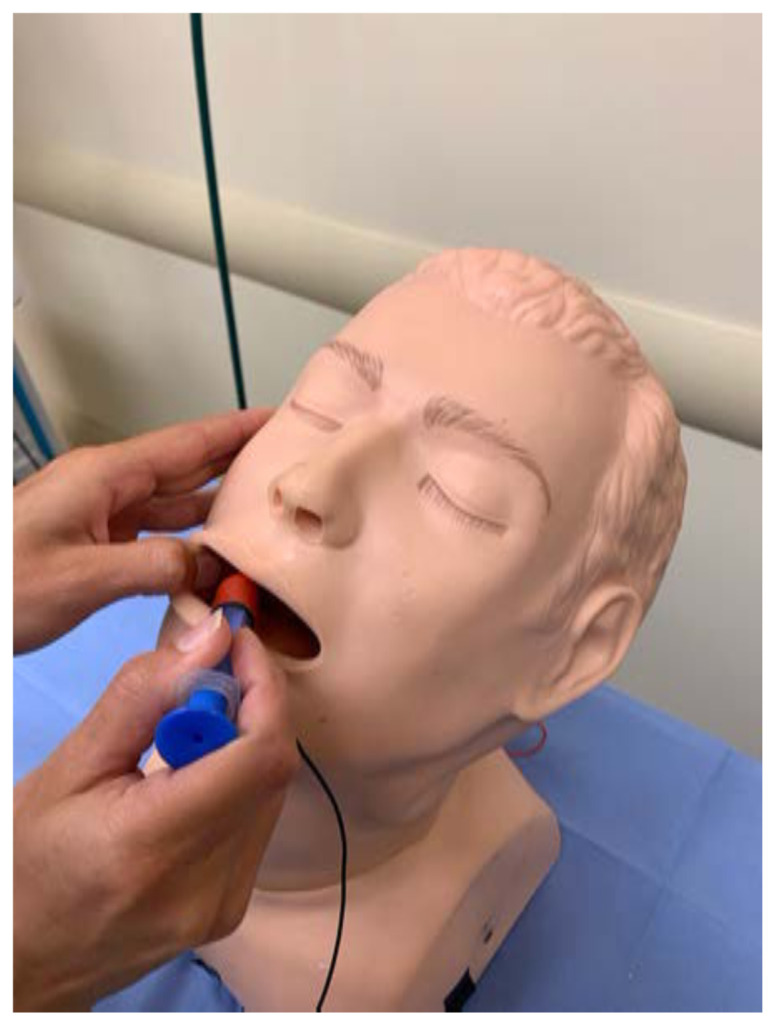

### Results and tips for successful implementation

This innovative partial task trainer has been successfully used as part of a simulation-based didactics program for PGY 1–4 EM residents. Following the session, learners were asked to complete a post-session survey. The survey contained basic demographic and procedural experience questions, Likert-type questions, and an open-response question. Twenty-one residents (10 PGY-1, 9 PGY-3, 2 PGY-4) participated in the didactic session and all completed the post-session survey. Many of the participants (N=10, 48%) had never previously performed these nerve blocks. On average, participants rated their comfort performing these specific nerve blocks before the session to be 1.96 on a 5-point Likert-scale (where 1=not at all comfortable and 5=extremely comfortable). Following the session, participants’ comfort increased to 3.67 on the same scale. Participants rated the usefulness of the feedback-enhanced task trainer to be a 4.71 on a 5-point scale (where 1=not at all useful and 5=extremely useful) and many provided additional positive comments, without recommendations for alterations or modifications.

### Discussion

Using inexpensive and commonly available materials, we were able to successfully modify an existing airway task trainer in order to create a feedback-enhanced partial task trainer for teaching regional anesthesia of the face and mouth. Learners reported that the educational session greatly increased their confidence in performing supra-orbital, infra-orbital, mental, and inferior alveolar nerve blocks. Additionally, they found the feedback-enhanced partial task trainer to be extremely helpful for teaching appropriate landmark identification. Our model was successfully used to teach facial and dental nerve block techniques which are able to provide both procedural anesthesia and non-opioid analgesia. Future studies could investigate whether this educational session and model leads to increased competence and/or increased performance of facial nerve blocks in the clinical setting.
